# Characterization of the fecal microbiome in cats with inflammatory bowel disease or alimentary small cell lymphoma

**DOI:** 10.1038/s41598-019-55691-w

**Published:** 2019-12-16

**Authors:** Sina Marsilio, Rachel Pilla, Benjamin Sarawichitr, Betty Chow, Steve L. Hill, Mark R. Ackermann, J. Scot Estep, Jonathan A. Lidbury, Joerg M. Steiner, Jan S. Suchodolski

**Affiliations:** 1 Gastrointestinal Laboratory, Department of Small Animal Clinical Science, Texas A&M University, 4474 TAMU, College Station, TX 77843-4474 USA; 2 University of California Davis, School of Veterinary Medicine, Department of Medicine & Epidemiology, Davis, CA 95616 USA; 3 Veterinary Specialty Hospital, 10435 Sorrento Valley Rd, San Diego, CA 92121 USA; 4 Oregon Veterinary Diagnostic Laboratory, Carlson College of Veterinary Medicine, Oregon State University, Corvallis, OR USA; 5 Texas Veterinary Pathology, LLC, San Antonio, TX USA; 6 Present Address: VCA Animal Specialty & Emergency Center, 1535 South Sepulveda Blvd, Los Angeles, CA 90025 USA; 7 Present Address: Flagstaff Veterinary Internal Medicine Consulting (FLG VIM-C), 6135 Kaitlin Way, Flagstaff, AZ 86003 USA

**Keywords:** Microbiome, Dysbiosis

## Abstract

Feline chronic enteropathy (CE) is a common gastrointestinal disorder in cats and mainly comprises inflammatory bowel disease (IBD) and small cell lymphoma (SCL). Both IBD and SCL in cats share features with chronic enteropathies such as IBD and monomorphic epitheliotropic intestinal T-cell lymphoma in humans. The aim of this study was to characterize the fecal microbiome of 38 healthy cats and 27 cats with CE (13 cats with IBD and 14 cats with SCL). Alpha diversity indices were significantly decreased in cats with CE (OTU p = 0.003, Shannon Index p = 0.008, Phylogenetic Diversity p = 0.019). ANOSIM showed a significant difference in bacterial communities, albeit with a small effect size (*P* = 0.023, R = 0.073). Univariate analysis and LEfSE showed a lower abundance of facultative anaerobic taxa of the phyla *Firmicutes* (families Ruminococcaceae and *Turicibacteraceae*), *Actinobacteria* (genus *Bifidobacterium*) and *Bacteroidetes* (i.a. *Bacteroides plebeius*) in cats with CE. The facultative anaerobic taxa *Enterobacteriaceae* and *Streptococcaceae* were increased in cats with CE. No significant difference between the microbiome of cats with IBD and those with SCL was found. Cats with CE showed patterns of dysbiosis similar to those in found people with IBD.

## Introduction

Feline chronic enteropathy (CE) is common in elderly cats and is defined as the presence of clinical signs of gastrointestinal disease for more than 3 weeks in the absence of infectious intestinal diseases (e.g., parasites) and extraintestinal causes (e.g., renal disease, hyperthyroidism)^1^.

Feline CE mainly comprises inflammatory bowel disease (IBD) and small cell lymphoma (SCL)^2–4^.

Diagnosis and differentiation can be challenging as clinical signs might be virtually the same in both disease entities^2,5^. A clinical diagnosis of IBD in cats is based on the presence of chronic gastrointestinal signs of at least 3 weeks duration, the absence of known enteropathogens or other causes of signs of gastrointestinal disease, and the histopathologic confirmation of intestinal inflammation^6^. Thus, the diagnosis of IBD and the differentiation from SCL requires histopathologic examination of tissue biopsies collected under general anesthesia^7,8^. In cases where histopathology results are ambiguous, additional diagnostic modalities such as immunohistochemistry and clonality testing are indicated^3,4,9,10^. Hence, the diagnosis of IBD and SCL is elaborate, expensive, time- and resource consuming and invasive. Treatment of IBD and SCL is usually based on immunosuppression using various steroids and cytotoxic medication such as chlorambucil or cyclosporine^2,6,11^. Therefore, less invasive diagnostic and treatment modalities would be highly desirable.

The intestinal microbiome plays a substantial role in modulating the host’s immune system within and beyond the gastrointestinal tract. Studies in people^12–14^ and dogs^15–17^ with IBD have found alterations in the composition of the intestinal microbiome that might impair the host’s health status. These changes are commonly referred to as dysbiosis^14^. Despite variations among studies, species, and individuals, common themes characterize intestinal dysbiosis. Across different species, three main hallmarks of dysbiosis have been described: a reduction in overall bacterial diversity (alpha diversity)^15,18–20^, a decreased stability of microbial communities and thus a higher fluctuation rate over time^21^, and a reduction in obligately anaerobic taxa of the phyla *Firmicutes* and *Bacteroidetes* at the expense of an increase in facultative anaerobes, including members of the family *Enterobacteriaceae*^15,17–19,21–24^. Dysbiosis has been described in people with various forms of enteropathy, including ulcerative colitis, Crohn’s disease, and colorectal cancer, and it might be a driver or consequence of chronic inflammation and malignant transformation^25^. Feline IBD and SCL share some features with chronic enteropathies in people. Both human and feline IBD is characterized by chronic inflammatory changes in the gastrointestinal tract^3,26^. Feline SCL is characterized by a monomorphic infiltration of the intestinal mucosa with small to medium lymphocytes, mostly in the small intestine and is often associated with epitheliotropism^4^. This histologic appearance resembles that of monomorphic epitheliotropic intestinal T-cell lymphoma (MEITL) in people, formerly known as enteropathy associated T-cell lymphoma Type 2 (EATL Type 2)^27,28^. Whereas SCL is quite common in cats, MEITL is rare in people, and thus large intersectional studies are scarce^28^. Both IBD and SCL occur spontaneously and frequently in cats, and thus the cat could be an interesting model for IBD or MEITL in people.

Several studies have characterized the fecal microbiome in cats with acute and chronic diarrhea^18,29^. However, in previous studies the cats were categorized based on clinical signs without confirmation of the underlying disease process. This study aimed to characterize and compare the fecal microbiome in healthy cats and cats with histopathologically confirmed CE (IBD or SCL).

## Results

### Animal demographics and clinical activity index

A total of 65 cats were enrolled into this study, 38 healthy cats and 27 cats with chronic enteropathy (13 with IBD and 14 with SCL). A fecal sample was collected from all cats. Demographic characteristics are shown in Table 1.

**Table 1:** Comparison of demographic data between healthy cats and cats with feline chronic enteropathy (FCE).

	Healthy	Feline CE	p value
number of cats	38	27	
median age in years (range)	9 (1–15)	10 (2–16)	0.052
median body weight in kg (range)	5.4 (2.5–8.6)	4.6 (2.9–10.5)	0.035
median body condition score (range)	5 (4–9)	4 (1–9)	<0.0001
sex	18 FS, 20 MN	11 FS, 16 MN	0.596
breeds	22 DSH, 4 DLH, 2 Maine Coon, 2 Persian, 1 Bombay, 1 Burmese, 1 DMH, 1 Norwegian Forest Cat, 1 Lynx, 1 mixed breed, 1 Sphinx	17 DSH, 3 DMH, 3 Siamese, 2 DLH, 1 Rag Doll, 1 mixed breed	

Abbreviations: FS female spayed, MN male neutered, DSH domestic shorthair, DLH domestic longhair, DMH domestic medium hair.

Age did not differ significantly between healthy cats (median age: 9 years, range: 1–15 years) and cats with CE (median age: 10 years, range: 2–16 years; P = 0.052). Cats with CE had a significantly lower body weight (median: 4.6 kg, range: 2.9–10.5 kg) and body condition score (BCS; median: 4, range: 1–9) than healthy cats (median body weight: 5.4 kg, range: 2.5–8.6 kg, median BCS: 5, range: 4–9; P = 0.035 and P < 0.001, respectively). Cats with SCL were significantly older (median age: 11.5 years, range: 7–16 years) than cats with IBD (median age: 8 years, range: 2–16; P = 0.028). Cats with IBD and cats with SCL did not show statistically significant differences with regard to sex (p = 0.816), body weight (p = 0.454), and BCS (p = 0.529). Cats with CE had a median feline chronic enteropathy activity index (FCEAI)^1^ score of 5 (range: 2–11). FCEAI did not differ between cats with IBD (median: 6, range: 3–11) and cats with SCL (median: 5, range: 2–10; p = 0.838).

### Sequence analysis and rarefaction

In total, the sequence analysis of the 65 fecal samples yielded 2,837,900 quality sequences (median per sample: 73,741; range: 43,660–145,373).

**Figure 1:**
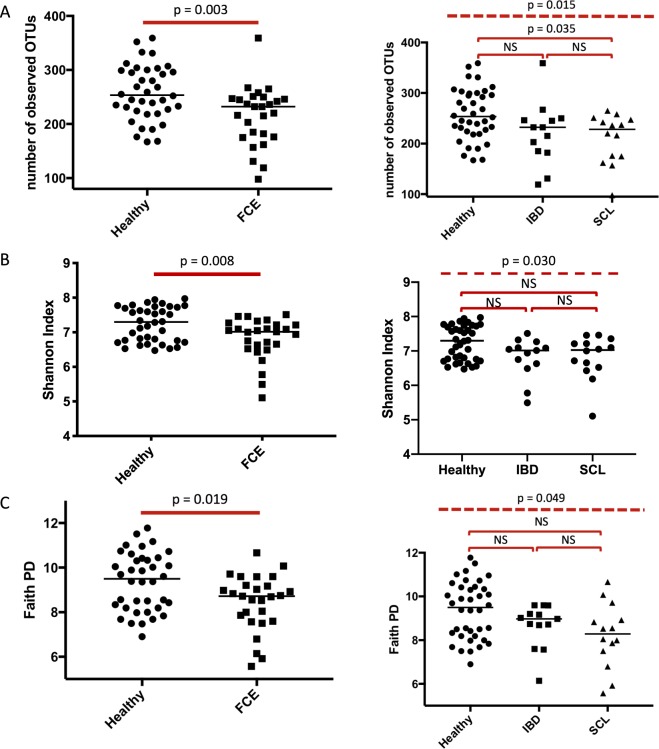
Alpha diversity indices at a depth of 43.660 sequences. (**A**) Observed OTUs. (**B**) Shannon Diversity Index. (**C**) Faith Phylogenetic Diversity Index. Figures on the left show the comparison between healthy cats and cats with chronic enteropathy (FCE). Figures on the right show the comparison between different the CE subgroups, inflammatory bowel disease (IBD) and small cell lymphoma (SCL). The top dashed line represents the Kruskal Wallis ANOVA comparison, solid parentheses represent post hoc multiple comparisons using Dunn’s test.

Alpha diversity at a depth of 43,660 sequences, as described by observed OTUs, Shannon Diversity Index, and Faith Phylogenetic Diversity Index, was significantly lower in cats with CE than in healthy cats (*P* = 0.003, *P* = 0.008, and *P* = 0.019, respectively; Fig. 1). In addition, alpha diversity appeared to continuously decrease from healthy cats, over cats with IBD to cats with SCL (Results for Kruskal Wallis ANOVA: observed OTUs P = 0.015, Shannon index P = 0.030, Phylogenetic Diversity Index P = 0.049). However, alpha diversity indices did not differ significantly between cats with IBD and cats with SCL. Detailed results for alpha diversity indices are shown in Supplementary Table 1.

### Microbial communities

**Figure 2:**
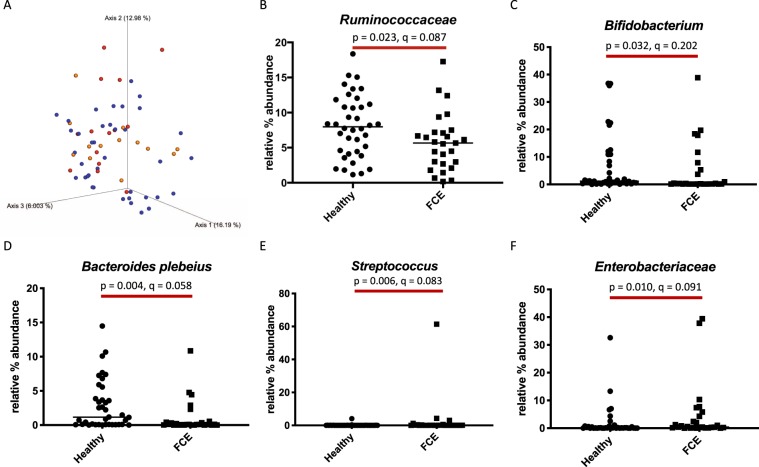
(**A**) Beta diversity. Principal coordinate analysis (PCoA) of unweighted UniFrac distances of 16S rRNA genes. Analysis of similarity (ANOSIM) revealed significantly different between healthy cats and cats with chronic enteropathy (*P* = 0.023, R = 0.073), although with a small effect size and no visible clustering. Healthy cats are depicted as blue, cats with inflammatory bowel disease (IBD) as yellow, and cats with small cell lymphoma (SCL) as red dots. (**B–F**) Univariate analysis of relative percent abundance of selected taxa. Members of the family *Ruminococcaceae*, of the genus *Bifidobacterium* and the species *Bacteroides plebeius* were found to be lower in cats with chronic enteropathy (CE) compared to healthy cats. Members of the genus *Streptococcus* and the family *Enterobacteriaceae* were found to be higher in cats with CE compared to healthy cats.

Although cats with CE showed no obvious visible clustering on Principal Component Analysis (PCoA) compared to healthy cats, a statistically significant difference between the two groups was found based on ANOSIM of unweighted Unifrac distances (*P* = 0.023, R = 0.073: Fig. 2A). Individual bacterial groups were analyzed using a Kruskal Wallis test. Taxa found to be significantly different before correction for the false discovery rate (FDR) are listed in Table 2. Within the phylum *Firmicutes*, bacterial taxa belonging to the family *Ruminococcaceae* (unclassified species of the genus *Oscillospira*) and members of the genus *Turicibacter* (class *Bacilli*, order *Turicibacterales*, family *Turicibacteraceae*) were significantly less abundant in cats with CE than in healthy cats. In addition, cats with CE had significantly decreased bacterial populations belonging to members of the phyla *Bacteroidetes* (one undetermined species and *Bacteroides plebeius*) and *Actinobacteria* (genus *Bifidobacterium*). In contrast, members of the families *Enterobacteriaceae* and *Streptococcaceae* were significantly more abundant in feces from cats with CE. However, although trends were noted, no statistically significant differences were found after correction for FDR. Figure 2B–F depicts results of the statistical analysis for some selected taxa.

**Table 2:** Taxa found to be significantly different (p value) between healthy cats and cats with chronic enteropathy (FCE) before correction for false discovery (q value). Numbers represent relative percentages.

Bacterial Group	Healthy		FCE		Healthy vs. FCE	
	Median	Range	Median	Range	Pvalue	Qvalue
**Class**						
Actinobacteria	1.2	0–36.8	0.2	0.1–38.9	0.0466	0.2563
Bacteroidia	25.3	1.2–56.4	16.6	0.7–55.3	0.0458	0.2563
**Order**						
Bifidobacteriales	1.2	0–36.8	0.2	0.1–38.9	0.0316	0.14885
Bacteroidales	25.3	1.2–56.4	16.6	0.7–55.3	0.0458	0.14885
Turicibacterales	0	0–23.7	0	0–5.8	0.0344	0.14885
Enterobacteriales	0.1	0–32.6	0.4	0–39.4	0.0098	0.1274
**Family**						
Bifidobacteriaceae	1.2	0–36.8	0.2	0.1–38.9	0.0316	0.16053333
Prevotellaceae	1.1	0–21.7	0.5	0–22.5	0.0519	0.1827
Odoribacteraceae	0	0–2.8	0.1	0–1.4	0.0522	0.1827
Paraprevotellaceae	0	0–18.5	0	0–5.6	0.0022	0.0616
Streptococcaceae	0	0–4.1	0.1	0–61.4	0.0059	0.0826
Turicibacteraceae	0	0–23.7	0	0–5.8	0.0344	0.16053333
Ruminococcaceae	8	1.2–18.4	5.7	0.2–17.3	0.0228	0.1596
Enterobacteriaceae	0.1	0–32.6	0.4	0–39.4	0.0098	0.09146667
**Genus**						
Bifidobacterium	1.2	0–36.8	0.2	0.1–38.9	0.0316	0.2021
Prevotella	0	0–18.5	0	0–5.6	0.0182	0.17108
Streptococcus	0	0–4.1	0.1	0–61.4	0.0037	0.08695
Turicibacter	0	0–23.7	0	0–5.8	0.0344	0.2021
Undetermined genus, Peptostreptococcaceae	0.3	0–16.6	0.1	0–5.9	0.04	0.20888889
Undetermined genus, Ruminococcaceae	0.1	0–0.9	0	0–0.5	0.0033	0.08695
Oscillospira	1.1	0.1–3.5	0.7	0–2.2	0.0181	0.17108
Undetermined genus, Erysipelotrichaceae	0.1	0–0.9	0	0–0.8	0.0241	0.18878333
Undetermined genus, Enterobacteriaceae	0.1	0–32.6	0.4	0–39.4	0.0081	0.1269
**Species**						
Undetermined species, Bifidobacterium	1.1	0–35.2	0.2	0–38.6	0.0234	0.13737
Undetermined species, Bacteroides	0.1	0–2.3	0	0–0.8	0.0041	0.058425
Undetermined species, Bacteroides	10.8	0.3–51	6.5	0.3–47.4	0.3514	0.5271
Bacteroides plebeius	1.2	0–14.5	0.1	0–10.9	0.0039	0.058425
Undetermined species, Prevotella	0	0–18.5	0	0–5.6	0.0182	0.129675
Undetermined species, Enterococcaceae	0.1	0–5	0	0–1.7	0.1968	0.37791
Undetermined species, Streptococcus	0	0–4.1	0.1	0–61.4	0.0109	0.10355
Undetermined species, Turicibacter	0	0–23.7	0	0–5.8	0.0344	0.1691
Undetermined species, Clostridium	0.2	0–10.8	0.9	0.1–20.3	0.0022	0.058425
Undetermined species, Ruminococcus	0	0–3.2	0.6	0–2.5	0.0356	0.1691
Undetermined species, Peptostreptococcaceae	0.3	0–16.6	0.1	0–5.9	0.04	0.17538462
Undetermined species, Ruminococcaceae	0.1	0–0.9	0	0–0.5	0.0033	0.058425
Undetermined species, Oscillospira	1.1	0.1–3.5	0.7	0–2.2	0.0181	0.129675
Undetermined species, Erysipelotrichaceae	0.1	0–0.9	0	0–0.8	0.0241	0.13737
Undetermined species, Enterobacteriaceae	0.1	0–32.6	0.4	0–39.4	0.0081	0.09234

Conversely, based on Linear Discriminant Analysis Effect Size (LEfSe), bacteria of the families *Bifidobacteriaceae*, *Ruminococcaceae*, *Turicibacteriaceae*, and *Paraprevotellaceae* were associated with feces from healthy cats, while *Enterobacteriaceae* and *Streptococcaceae* were associated with those of cats with CE. A detailed summary of the LEfSe results can be found in Table 3.

**Table 3:** Linear discriminant analysis effect size (LEfSE) analysis of 16S rRNA gene sequences.

Level	Selected Taxa	Associated Group	LDA Score	Associated Subgroup	LDA Score
**Phylum**	Bacteroidetes	Healthy	4.797	None	NA
**Class**	Actinobacteria	Healthy	4.562	Healthy	4.662
	Bacteroida	Healthy	4.74	None	NA
**Order**	Bifiodobacteriales	Healthy	4.196	Healthy	4.659
	Turicibacterales	Healthy	4.356	None	NA
	Enterobacteriales	FCE	4.302	None	NA
	Bacteroidales	Healthy	4.549	None	NA
**Family**	Streptococcaceae	FCE	4.229	SCL	4.425
	Ruminococcaceae	Healthy	4.204	None	NA
	Bifidobacteriaceae	Healthy	4.227	Healthy	4.394
	Enterobacteriaceae	FCE	4.256	None	NA
	Paraprevotellaceae	Healthy	4.009	Healthy	4.116
	Turicibacteraceae	Healthy	4.04	None	NA
	Odoribacteraceae	None	NA	SCL	4.289
	Prevotellaceae	None	NA	Healthy	3.851
**Genus**	Erysipelotrichaceae	Healthy	3.672	None	NA
	Oscillospira	Healthy	3.534	None	NA
	Peptostreptococcaceae (unclassified)	Healthy	3.594	None	NA
	Bifidobacterium	Healthy	4.293	Healthy	4.496
	Turicibacter	Healthy	3.632	None	NA
	Streptococcus	FCE	4.119	SCL	4.511
	Paraprevotella	Healthy	3.789	None	NA
	Enterobacteriaceae (unclassified)	FCE	4.226	None	NA
	Prevotella	Healthy	4.109	Healthy	4.304
	Coriobacteriaceae (unclassified)	None	NA	IBD	4.187
**Species**	Turicibacter (unclassified)	Healthy	3.578	None	NA
	Streptococcus (unclassified)	FCE	4.092	SCL	4.494
	Oscillospira (unclassified)	Healthy	3.509	None	NA
	Bacteroides plebeius	Healthy	4.069	Healthy	4.285
	Enterobacteriaceae (unclassified)	FCE	4.255	None	NA
	Paraprevotella (unclassified)	Healthy	3.465	None	NA
	Peptostreptococcaceae (unclassified)	Healthy	3.652	None	NA
	Bifidobacterium (unclassified)	Healthy	3.972	None	NA
	Erysipelotrichaceae (unclassified)	Healthy	3.626	None	NA
	Prevotella copri	None	NA	Healthy	4.258
	Coriobacteriaceae (unclassified)	None	NA	SCL	4.169

LEfSE was calculated for healthy cats vs. cats with chronic enteropathy (FCE) and in a second step for the subgroups of FCE, inflammatory bowel disease (IBD) and small cell lymphoma (SCL). The Linear Discriminant Analysis Score (LDA) is given as log 10. Only the taxa meeting a significant LDA threshold value of >2 are shown.

### Effect of disease subtype on the feline fecal microbiota

Similar to alpha-diversity, beta-diversity showed continuous changes when comparing healthy cats, cats with IBD, and cats with SCL, with sequential increases or decreases of relative percentages between groups. According to Kruskal Wallis tests, the abundance of members of the genus *Bifidobacterium* (class *Actinobacteria*) differed significantly among the three groups, with highest numbers in healthy cats and lowest numbers in cats with SCL. In contrast, bacteria within the families *Enterobacteriaceae* (phylum *Proteobacteria*) and *Streptococcaceae* (phylum *Firmicutes*) serially increased from healthy cats, to cats with IBD to cats with SCL. However, after correction for the false discovery rate (FDR), none of the differences remained statistically significant. In addition, no significant differences in microbial communities between cats with IBD and cats with SCL were observed. A detailed summary of relative percentages of the most abundant bacterial groups appears in Supplementary Table 2.

## Discussion

To our knowledge, this is the first study comparing the fecal microbiome in a cohort of cats with histopathologically confirmed spontaneous CE to that of clinically healthy cats using untargeted Illumina sequencing analysis. Alpha diversity was significantly lower in cats with CE than in healthy cats. In particular, cats with CE tended to show a lower abundance of obligately anaerobic members of the phyla Firmicutes (family *Ruminococcaceae* and *Turicibacteraceae*), Bacteroidetes (e.g. *Bacteroides plebeius* and unclassified species), and Actinobacteria (genus *Bifidobacterium)*. In contrast, facultative anaerobes such as *Enterobacteriaceae* and *Streptococcaceae* tended to be more abundant in cats with CE than in healthy cats. However, although we found differences in abundance of various bacterial taxa between different groups of cats, there were no statistically significant differences after correction for FDR. Nevertheless, the trends found in our cohort of cats mirror common patterns of fecal and mucosal dysbiosis described in other species such as humans^19,21–24^ and dogs^15,17,30^, i.e. decreased bacterial diversity, decreased members of obligate anaerobes (*Firmicutes* and *Bacteroidetes*) and increased facultative anaerobes (especially of the family of *Enterobacteriaceae*). Dysbiosis has been documented in humans with various enteropathies, such as IBD (ulcerative colitis and Crohn’s disease), antibiotic-associated diarrhea, necrotizing enterocolitis, and colorectal cancer^14,31^ and also in dogs with chronic enteropathies^16,30,32^. Dysbiotic patterns appear to be similar across different forms of enteropathy; however, because the rarity of the disease, there are no studies published on the microbiomes of people with MEITL. A recent study analyzing the microbiome in feces from dogs with IBD or intestinal lymphoma compared to healthy dogs found dysbiosis to be associated with both disease entities^32^. Similar to the current study, the authors used an untargeted Illumina sequencing approach to characterize the fecal microbiome. The authors reported an increased abundance of *Coprococcus, Oscillospira*, and *Eubateria* in dogs with intestinal lymphoma compared to healthy dogs. These findings might indicate species differences in disease phenotype and etiopathogenesis.

One universal activity of the intestinal microbiome is the metabolism and fermentation of carbohydrates into short chain fatty acids, such as acetate, butyrate, and propionate. Members of the phylum *Firmicutes* are mostly anaerobic and have been shown to exert indirect anti-inflammatory and immune-modulatory effects by producing short chain fatty acids, particularly butyrate^14^. Butyrate is the major energy source of colonocytes^33^, thereby contributing to epithelial cell proliferation and repair and to intestinal barrier integrity^34^. In addition, evidence exists that butyrate may exhibit anti-inflammatory and anti-carcinogenic properties^35,36^. The main butyrate-producing bacteria in the human colon are members of the families *Lachnospiraceae* and *Ruminococcaceae* (phylum *Firmicutes*), and their fecal abundance in people is often depleted in dysbiotic states, including UC and CD^21,37^. Our report appears to be the first to show a depletion of butyrate-producing bacteria in cats with CE. Determining the association between the fecal abundance of *Ruminococcaceae* and butyrate concentration in cats with CE might further elucidate the function of this taxon in cats with CE.

Another major hallmark of dysbiosis involves members of the phylum *Bacteroidetes*. *Bacteroidetes* are highly abundant in the healthy microbiome but are decreased in the mucosal or fecal microbiome from humans and dogs with various forms of enteropathy^17,19,22^. Interestingly, *Bacteroides plebeius* has been found to be strongly associated with remission in people with CD^38^. In our cohort, *Bacteroidetes* and specifically *Bacteroides plebeius* tended to be decreased in cats with CE. Investigating the association between disease outcome and abundance of *Bacteroides plebeius* might be of interest for future studies in cats with CE.

Our study showed a trend towards a decreased abundance of Bifidobacteria in cats with IBD and SCL. Bifidobacteria are also commonly decreased in fecal and mucosal samples from human patients with IBD^14,39^. Various Bifidobacteria strains have been shown to exert anti-inflammatory properties by regulating immune cells and cytokine networks^40^ and by directly and indirectly enhancing intestinal barrier function^41,42^. For instance, *Bifidobacterium* strains have been shown to induce IL-10 producing regulatory T-cells^43,44^ and to exert immunoinhibitory effects by interacting with Toll-like receptor-2^45^. Therefore, *Bifidobacteria* have become an attractive therapeutic target and are often used in probiotic formulations^46^. Our study confirms findings of a previous study utilizing fluorescence *in situ* hybridization, where *Bifidobacteriaceae* were reported to be decreased in fecal samples in cats with IBD. To the authors’ knowledge, such a decrease has not been documented in dogs. This highlights the difference between species and might point toward *Bifidobacteria* as potential therapeutic targets in cats with CE.

Besides a decrease in obligate anaerobic bacteria, dysbiosis is commonly characterized by an increase in facultative anaerobic bacteria, specifically members of the family of *Enterobacteriaceae*^14,47,48^. This phenomenon might be explained by the oxygen gradient model^49,50^. In this model it is hypothesized that during a steady state, the mucosal microbiome is controlled by mucosal immune responses, the intestinal barrier, and competition with luminal bacteria. The intestinal mucosa shows an oxygen gradient, in which the mucosal interface is mostly aerobic while the lumen is mostly anaerobic. It is thought that during inflammation, the intestinal barrier breaks downs, thereby increasing the luminal oxygen tension. This leads to a translocation and expansion of aerotolerant taxa usually located close to the mucosa centripetal into the lumen and centrifugal across the epithelial barrier into the lamina propria, contributing to the inflammatory response^49,50^. Our cohort of cats followed this pattern, with a trend of higher abundance of facultative anaerobic taxa, specifically *Enterobacteriaceae* and *Streptococcaceae*. Both taxa typically consist of facultative anaerobic members and thus might thrive with increased luminal oxygen tension. An increased abundance of *Streptococcaceae* has previously been documented in feces from people and dogs with IBD^51,52^.

Our study has several limitations. With regards to enrollment criteria, of 38 control cats enrolled into this study, 22 cats underwent a physical examination and laboratory testing to verify their health status, while 16 cats were classified as healthy based on owner responses to a questionnaire alone. In addition, a clinical diagnosis of IBD was based on the presence of chronic gastrointestinal signs of at least 3 weeks duration, the absence of known enteropathogens or other causes of signs of gastrointestinal disease, and the histopathologic confirmation of intestinal inflammation^6^. Thus, food-responsive enteropathy was not excluded in all of the enrolled cats. However, many human patients with IBD show complete or partial responses to dietary interventions without being reclassified as having food-responsive enteropathy^53–56^. Our study aimed to characterize clinically relevant differences between the fecal microbiome of cats with CE when compared to healthy control cats. We controlled for demographic characteristics such as age, sex, and breed. Environmental factors, such as housing, diet, previous parasitic or systemic infections etc. were not controlled for in this study, as it would have affected the clinical relevance of the results. This concept is supported by recent studies showed that standardization in is a major source of poor reproducibility of preclinical trials^57,58^.

With regards to results of this study, the following limitations have to be acknowledged. Although, we found differences in the abundance of various bacterial taxa between different groups of cats, there were no statistically significant differences after correction for FDR, and thus differences did not appear to be as strong as they have been reported for humans and dogs. This phenomenon might be explained by the different disease phenotypes. Whereas diarrhea is the predominant clinical sign of chronic enteropathy in humans^59^ and dogs^60,61^, it is less common in cats, in which weight loss, hyporexia, and vomiting are the dominant clinical signs of CE^1,2^. In our cohort of cats, only 5 out of 27 cats showed diarrhea. The reason for the different disease phenotypes in humans, dogs, and cats is not entirely clear, but factors may include different disease localization (i.e. small vs. large bowel) and different disease pathophysiology. Cats often show involvement of the small intestinal tract^1,2,4^, but the fecal microbiome represents predominantly bacterial communities present in the distal part of the intestinal tract^62^. In addition, the fecal microbiome might not accurately represent the mucosa-associated microbiome^62^. Therefore, investigating the luminal content within the small intestine or the mucosal microbiome might reveal more distinct differences in microbial communities in cats than this study of fecal samples. However, a recent study comparing the fecal and mucosal microbiomes in human patients with and without IBD found large overlaps between the two microbial habitats in each group^49^. Although it is important to point out that IBD in humans is predominantly a large bowel disease. A recent study, comparing the mucosa associated microbiome in cats with CE using targeted analysis by fluorescence *in situ* hybridization found significantly increased numbers of *Bacteroides spp*. in ileal biopsies from cats with SCL compared to biopsies from cats with IBD^63^. These findings further underline possible differences between intestinal compartments (small vs. large bowel) and media (e.g. fecal vs mucosa-associated microbiome). We also cannot exclude that a larger sample size would have revealed statistically significant differences after FDR correction. Finally, the mode and speed of fecal collection and processing was slightly different between groups and individuals and might have had an effect on our results.

Differentiation of IBD from SCL in cats can be challenging as clinical signs as well as intestinal mucosal changes can be overlapping^3^. In addition, mucosal changes can have an uneven distribution, inflammation and neoplastic lesions can coexist might and it has been suggested that inflammatory lesions might progress to lymphoma over time^3,4^. Hence, every classification into groups of IBD and SCL is associated with a degree of uncertainty. However, all cats in the CE group underwent gastoduodenoscopy and ileoscopy. In addition, immunohistochemistry and clonality testing were applied, where indicated, to ensure the maximum accuracy ion class assignment.

Today, histopathology, immunohistochemistry and clonality testing on intestinal samples is considered to be the gold standard for the differentiation of inflammatory from neoplastic lesions^10^. However, histopathological examination of intestinal tissue biopsies from dogs and cats is associated with a high inter-observer variability^64^. In addition, a recent study showed that healthy elderly cats can also have abnormal histopathological findings without any apparent clinical significance^65^. Therefore, alternative methods for the diagnosis and differentiation of feline IBD and SCL would be desirable. We did not find any bacterial taxa in which abundance differed significantly between cats with IBD and SCL. However, as a striking pattern many bacterial taxa appeared to either serially increase (e.g., *Enterobacteriaceae*) or decrease (e.g., *Actinobacteria, Bifidobacteria, Prevotella*) when comparing to healthy cats, cats with IBD, and cats with SCL. Similarly, progression of IBD to SCL over months to years has long been suspected, and inflammatory lesions frequently coexist with SCL^4,9^. Our findings might lend support to the hypothesis that IBD and SCL are not two different diseases but rather a continuum.

In summary, we found that cats with CE show patterns of dysbiosis that have previously been described in people with IBD. Obligately anaerobic taxa in the phyla Firmicutes, Bacteroidetes, and Actinobacteria were depleted in cats with CE, while facultative anaerobes such as *Enterobacteriaceae* and *Streptococcaceae* were more abundant. *Bacteroides plebeius*, a species shown to be associated with positive outcome in people with CD, was decreased in our cohort of cats.

## Methods

### Animals

This prospective study was conducted at the Veterinary Medical Teaching Hospital at Texas A&M University between May 2015 and September 2017. The study protocol was approved by the Texas A&M University Institutional Animal Care and Use Committee and all methods were performed in accordance with relevant guidelines and regulations.

The health status of cats in the group considered healthy was verified by an owner questionnaire on general and gastrointestinal health. The questionnaire covered the following areas: attitude/activity, appetite, drinking, urination, chronic illnesses, weight loss, vomiting, diarrhea, and treatment with antibiotics, antacids, anti-inflammatory drugs, or steroids. In 22 cats, physical examination was performed by a single board-certified internist (SM). The body condition score was assessed using a previously established a nine-point condition scoring system^66^. Blood was collected from a peripheral vein or the jugular vein and the following tests were performed: complete blood count, serum chemistry profile, total T4, cobalamin, folate, feline pancreatic lipase immunoreactivity (fPLI), and feline trypsin-like immunoreactivity (fTLI). Cats with gastrointestinal signs (weight loss, hyporexia, vomiting > 2x/ month, diarrhea) within 6 months prior to enrollment were excluded. In addition, cats with systemic diseases, chronic illnesses or clinically significant laboratory abnormalities were excluded from the study. Finally, cats that had received any antibiotics, antacids, anti-inflammatory drugs, or corticosteroids within the past 6 months were excluded.

Cats with clinical signs of chronic enteropathy (weight loss, hyporexia, vomiting, diarrhea) of at least 3 weeks duration were eligible for enrollment into the CE group. Cats in this group were either presented to the Small Animal Hospital at Texas A&M University, College Station, Texas, or the Veterinary Specialty Hospital, San Diego, California. Extra-gastrointestinal disease as well as possible infectious intestinal diseases were excluded based on a complete blood count, serum chemistry profile, total T4 and fecal examination. All cats in this group underwent gastro-duodenoscopy and ileo-colonoscopy for diagnostic purposes. Histopathologic examination of H&E stained endoscopic formalin-fixed, paraffin-embedded (FFPE) tissue sections was performed by board-certified pathologists (MA or JSE) blinded to the clinical status of the cats.

Cases with a histopathological diagnosis of SCL or in which the pathologist was suspicious of an underlying SCL underwent additional diagnostic testing with immunohistochemistry and PCR for antigen receptor rearrangement testing for diagnostic confirmation. A final diagnosis of IBD or SCL was reached upon integration of results from histopathology, immunohistochemistry, and PARR based on the current EuroClonality/BIOMED-2 guidelines for interpretation and reporting of Ig/TCR clonality testing in suspected lymphoproliferations^10,67,68^. Cats that had received antibiotics within 4 weeks or corticosteroids within the past 2 weeks prior to fecal sampling were excluded from the study.

Spontaneously passed fecal samples were collected from healthy cats, refrigerated and shipped to the Texas A&M Gastrointestinal Laboratory within 24 hours of passing. Fecal samples from cats with CE were either collected after spontaneous void or digitally while the cat was under anesthesia for endoscopy. All samples were shipped on cold packs or on dry ice. Upon arrival, fecal samples were immediately transferred to a lysis buffer and DNA was extracted using the Mobio Power Soil DNA Extraction kit (MoBio Laboratories, Inc., CA) following manufacturer’s instructions.

Amplification and sequencing of the V4 variable region of the 16 S rRNA gene were performed utilizing the Illumina MiSeq Sequencing platform. Sequencing was performed at MR DNA (Shallowater, TX, USA) following the manufacturer’s guidelines using forward and reverse primers: 515 F (5′-GTGCCAGCMGCCGCGGTAA-3′) and 806 R (5′- GGACTACVSGGGTATCTAAT-3′). Briefly, the PCR reaction was performed in a single-step 28 cycle PCR using the HotStarTaq Plus Master Mix Kit (Qiagen, USA) under the following conditions: 94 °C for 3 minutes, followed by 28 cycles (5 cycles used on PCR products) of 94 °C for 30 seconds, 53 °C for 40 seconds and 72 °C for 1 minute, after which a final elongation step at 72 °C for 5 minutes was performed. After sequencing, barcodes and primers were removed from the sequences; then short (<150 bp), ambiguous, homopolymeric, and chimeric sequences were depleted from the dataset. Operational Taxonomic Units (OTUs) were assigned based on at least 97% sequence similarity using the QIIME 2.0 pipeline^69^. Sequences assigned as chloroplast, mitochondria and Unassigned were removed before downstream analysis. Additionally, OTUs assigned to the phylum cyanobacteria were considered to be potential plant chloroplast contaminants and excluded from the analysis. Sequences were rarefied to an equal depth of 12,000 sequences per sample. The sequences were deposited in SRA under accession number SRP168128.

### Statistical analysis

All datasets were tested for normality using the Shapiro-Wilk test (JMP 10, SAS software Inc.). Differences in bacterial communities between healthy cats and cats with CE were analyzed using the phylogeny-based unweighted UniFrac distance metric, and PCoA plots and rarefaction curves were generated within QIIME^69^. ANOSIM (Analysis of Similarity) within the software package PRIMER 6 (PRIMER-E Ltd., Luton, UK) was used to determine significant differences in microbial communities between healthy cats and diseased cats. Because most datasets did not meet the assumptions of normal distribution, statistical testing between healthy and diseased cats were performed using non-parametric Kruskal-Wallis tests or a Mann-Whitney U test where applicable. The resulting p-values were adjusted for multiple comparisons using the Benjamini & Hochberg’s False Discovery Rate (FDR), and an adjusted q < 0.05 was considered statistically significant^70^. A Dunn’s post-test was used to determine which disease types differed significantly. Linear discriminant analysis Effect Size (LEfSe) was used to elucidate bacterial taxa (16 S rRNA genes) associated with healthy or diseased cats. LEfSe was used online in the Galaxy workflow framework.

The data that support the findings of this study are available from the corresponding author (SM) upon reasonable request.

### Ethical approval and informed consent

The study protocol was approved by the Texas A&M University Animal Care and Use Committee (IACUC 2015-0276 CA and IACUC 2014-0369 CA).

## Supplementary information

Supplementary Table 1 and 2.
