# Supplementary material for: Characterization of the fecal microbiome in cats with inflammatory bowel disease or alimentary small cell lymphoma
Source: Sci Rep. 2019 Dec 16;9:19208. doi: 10.1038/s41598-019-55691-w (PMC6914782; doi:10.1038/s41598-019-55691-w)
Supplement: Supplementary file 1 — Supplementary Table 1 and 2. [file 41598_2019_55691_MOESM1_ESM.pdf]

Characterization of the fecal microbiome in cats with inflammatory bowel disease or alimentary small cell lymphoma

S Marsilio[1, 2*], R Pilla[1], B Sarawichitr[1], B Chow[3], S Hill[3], MR Ackermann[4], JS Estep[5], JA Lidbury[1], JM Steiner[1], JS Suchodolski[1]

1. Gastrointestinal Laboratory, Department of Small Animal Clinical Science, Texas A&M University, 4474 TAMU, College Station, TX 77843-4474, USA 2. University of California Davis School of Veterinary Medicine and Epidemiology, Davis, CA 95616, USA 3. Veterinary Specialty Hospital, 10435 Sorrento Valley Rd, San Diego, CA 92121, USA, 4. Oregon Veterinary Diagnostic Laboratory, Carlson College of Veterinary Medicine, Oregon State University, Corvallis, OR. 5. Texas Veterinary Pathology, LLC., San Antonio, TX

Dr. B Chow's current affiliation is the VCA Animal Specialty & Emergency Center 1535 S Sepulveda Blvd, Los Angeles, CA 90025. Dr. S Hill's current affiliation is Ethos Diagnostic Science by Ethos Veterinary Health, 10455 Sorrento Valley Road Suite 100 San Diego, CA 92121.

*corresponding author: SMarsilio@ucdavis.edu

Index	Healthy	FCE		subgroup FCE-IBD	subgroup FCE - SCL	Kruskal Wallis ANOVA p value	Dunn's post hoc p value		
	median (range)	median (range)	p value	median (range)	median (range)		H/IBD	H/SCL	IBD/SCL
**OTUs**	253.5 (167 – 359)	232 (98 – 359)	0.003	232 (119 – 359)	228 (98 – 265)	0.015	0.120	0.035	>0.999
**Shannon**	7.3 (6.5 – 8.0)	7.0 (5.1 – 7.5)	0.008	7.0 (5.5 – 7.5)	7.0 (5.1 – 7.5)	0.030	0.100	0.112	>0.999
**Faith PD**	9.5 (6.9 – 11.8)	8.7 (5.6 – 10.7)	0.019	9.0 (6.1 – 9.6)	8.3 (5.6 – 10.7)	0.049	0.500	0.061	>0.999

Supplementary Table 1. Summary of alpha diversity indices at a depth of 43.660 sequences per sample comparing healthy cats and cats with CE (FCE) with subgroups inflammatory bowel disease (IBD) and small cell lymphoma (SCL).

Abbreviations: FCE Feline chronic enteropathy, OTUs observed operational taxonomic units, STD Standard deviation, PD Phylogenetic Index, H Healthy cats

Bacterial Group	Healthy		IBD		SCL		Healthy vs IBD vs SCL[1]		Healthy vs IBD[2]		Healthy vs SCL[2]		IBD vs SCL[2]	
	Median	Range	Median	Range	Median	Range	Pvalue	Qvalue	Pvalue	Qvalue	Pvalue	Qvalue	Pvalue	Qvalue
**Class**														
Actinobacteria	1.2	0-36.8	0.4	0.2-38.9	0.2	0.1-18.4	0.0336	0.359975	0.9999	0.9999	0.0281	0.3091	0.2787	0.9999
Gammaproteobacteria	0.7	0-32.6	0.5	0-39.5	1.8	0.2-37.8	0.0727	0.359975	0.9999	0.9999	0.1007	0.55385	0.1545	0.9999
**Order**														
Bifidobacteriales	1.2	0-36.8	0.4	0.2-38.9	0.2	0.1-18.4	0.0297	0.19305	0.9999	0.9999	0.024	0.156	0.3609	0.9999
Turicibacterales	0	0-23.7	0	0-5.8	0	0-0.1	0.0738	0.3198	0.7844	0.9999	0.0808	0.350133	0.9999	0.9999
Enterobacteriales	0.1	0-32.6	0.2	0-39.4	1	0-37.8	0.0187	0.19305	0.5643	0.9999	0.0177	0.156	0.7669	0.9999
**Family**														
Bifidobacteriaceae	1.2	0-36.8	0.4	0.2-38.9	0.2	0.1-18.4	0.0297	0.16632	0.9999	0.9999	0.024	0.2366	0.3609	0.9999
Prevotellaceae	1.1	0-21.7	0.6	0.2-18.8	0.1	0-22.5	0.0365	0.170333	0.9999	0.9999	0.0309	0.2366	0.2749	0.9999
Odoribacteraceae	0	0-2.8	0	0-1.3	0.8	0-1.4	0.0093	0.1302	0.9999	0.9999	0.0091	0.2366	0.0545	0.9999
Paraprevotellaceae	0	0-18.5	0	0-0.3	0	0-5.6	0.0092	0.1302	0.061	0.4928	0.0338	0.2366	0.9999	0.9999
Streptococcaceae	0	0-4.1	0.1	0-4.3	0.1	0-61.4	0.0221	0.1547	0.0704	0.4928	0.103	0.480667	0.9999	0.9999
Ruminococcaceae	8	1.2-18.4	5.8	0.3-9.8	5.6	0.2-17.3	0.0532	0.19425	0.0647	0.4928	0.5395	0.9999	0.9999	0.9999
Enterobacteriaceae	0.1	0-32.6	0.2	0-39.4	1	0-37.8	0.0187	0.1547	0.7382	0.9999	0.495	0.9999	0.9999	0.9999
**Genus**														
Bifidobacterium	1.2	0-36.8	0.4	0.2-38.9	0.2	0.1-18.4	0.0297	0.2376	0.9999	0.9999	0.024	0.4944	0.3609	0.9999
Undetermined genus, Coriobacteriaceae	0.1	0-16.4	0.4	0-11.3	0.1	0-7.2	0.0258	0.2376	0.453	0.9999	0.1991	0.735138	0.0216	0.9999
Prevotella	1.1	0-21.7	0.6	0.2-18.8	0.1	0-22.5	0.0365	0.250286	0.9999	0.9999	0.0309	0.4944	0.2749	0.9999
Odoribacter	0	0-2.8	0	0-1.3	0.3	0-1.4	0.0513	0.280985	0.9999	0.9999	0.0472	0.5664	0.2684	0.9999
Streptococcus	0	0-4.1	0.1	0-4.3	0.1	0-61.4	0.0145	0.2376	0.0538	0.6456	0.0727	0.634286	0.9999	0.9999
Peptococcus	0.4	0-3	0.4	0-2.8	0.3	0-2	0.9571	0.9571	0.9999	0.9999	0.9999	0.9999	0.9999	0.9999
Undetermined genus, Peptostreptococcaceae	0.3	0-16.6	0.1	0-1	0.1	0-5.9	0.1208	0.35304	0.2844	0.9999	0.3327	0.9999	0.9999	0.9999
Undetermined genus, Ruminococcaceae	0.1	0-0.9	0	0-0.4	0	0-0.5	0.0126	0.2376	0.0356	0.6456	0.0925	0.634286	0.9999	0.9999
Oscillospira	1.1	0.1-3.5	0.4	0-1.5	0.9	0-2.2	0.0245	0.2376	0.0212	0.6456	0.8161	0.9999	0.5258	0.9999
Undetermined genus, Enterobacteriaceae	0.1	0-32.6	0.2	0-39.4	1	0-37.8	0.0159	0.2376	0.5065	0.9999	0.0153	0.4944	0.7822	0.9999

Continued Supplementary Table 2

Bacterial Group	Healthy		IBD		SCL		Healthy vs IBD vs SCL[1]		Healthy vs IBD[2]		Healthy vs SCL[2]		IBD vs SCL[2]	
	Median	Range	Median	Range	Median	Range	Pvalue	Qvalue	Pvalue	Qvalue	Pvalue	Qvalue	Pvalue	Qvalue
**Species**														
Undetermined species, Bifidobacterium	1.1	0-35.2	0.4	0-38.6	0.1	0-11.5	0.047	0.259253	0.249	0.9617	0.0632	0.529689	0.9999	0.9999
Undetermined species, Coriobacteriaceae	0.1	0-16.4	0.4	0-11.3	0.1	0-7.2	0.0258	0.217457	0.453	0.9999	0.1991	0.734181	0.0216	0.9999
Undetermined species, Bacteroides	0.1	0-2.3	0	0-0.8	0	0-0.5	0.0093	0.18762	0.34	0.9999	0.0098	0.45135	0.8596	0.9999
Bacteroides plebeius	1.2	0-14.5	0.1	0-10.9	0.1	0-4.8	0.0143	0.18762	0.1414	0.641738	0.0296	0.464133	0.9999	0.9999
Prevotella copri	1.1	0-21.7	0.6	0.2-18.8	0.1	0-22.5	0.0462	0.259253	0.9999	0.9999	0.0411	0.464133	0.2789	0.9999
Undetermined species, Odoribacter	0	0-2.8	0	0-1.3	0.3	0-1.4	0.0513	0.259253	0.9999	0.9999	0.0472	0.464133	0.2684	0.9999
Undetermined species, Streptococcus	0	0-4.1	0.1	0-4.3	0.1	0-61.4	0.0329	0.242638	0.0549	0.6195	0.267	0.875167	0.9999	0.9999
Undetermined species, Turicibacter	0	0-23.7	0	0-5.8	0	0-0.1	0.0738	0.259253	0.7844	0.9999	0.0808	0.529689	0.9999	0.9999
Undetermined species, Clostridiales	1	0-4.7	0.5	0-5.1	1.3	0-5.1	0.0555	0.259253	0.0486	0.6195	0.9999	0.9999	0.405	0.9999
Undetermined species,Clostridium	0.2	0-10.8	0.9	0.1-4.2	0.8	0.1-20.3	0.0093	0.18762	0.0457	0.6195	0.0452	0.464133	0.9999	0.9999
Undetermined species,Ruminococcaceae	0.1	0-0.9	0	0-0.4	0	0-0.5	0.0126	0.18762	0.0356	0.6195	0.0925	0.54575	0.9999	0.9999
Undetermined species, Oscillospira	1.1	0.1-3.5	0.4	0-1.5	0.9	0-2.2	0.0245	0.217457	0.0212	0.6195	0.8161	0.9999	0.5258	0.9999
Undetermined species, Enterobacteriaceae	0.1	0-32.6	0.2	0-39.4	1	0-37.8	0.0159	0.18762	0.5065	0.9999	0.0153	0.45135	0.7822	0.9999

Supplementary Table 2. Taxa found to be significantly different (p value) between healthy cats, cats with inflammatory bowel disease (IBD), and cats small cell lymphoma (SCL) before correction for false discovery (q value). Numbers represent relative percentages. Legend: [1] Kruskal Wallis ANOVA, [2] Dunn's post hoc test
